# Cost-effectiveness of meglumine antimoniate versus miltefosine caregiver DOT for the treatment of pediatric cutaneous leishmaniasis

**DOI:** 10.1371/journal.pntd.0005459

**Published:** 2017-04-06

**Authors:** Brandon A. Berger, Alexandra Cossio, Nancy Gore Saravia, Maria del Mar Castro, Sergio Prada, Allison H. Bartlett, Mai T. Pho

**Affiliations:** 1 University of Chicago Pritzker School of Medicine, Chicago, Illinois, United States of America; 2 Centro Internacional de Entrenamiento e Investigaciones Médicas (CIDEIM), Cali, Valle de Cauca, Colombia; 3 PROESA, Universidad Icesi, Cali, Valle de Cauca, Colombia; 4 Department of Pediatrics, Section of Infectious Diseases, University of Chicago Medicine, Chicago, Illinois, United States of America; 5 Department of Medicine, Section of Infectious Diseases and Global Health, University of Chicago, Chicago, Illinois, United States of America; Institute of Tropical Medicine, BELGIUM

## Abstract

**Background:**

Oral miltefosine has been shown to be non-inferior to first-line, injectable meglumine antimoniate (MA) for the treatment of cutaneous leishmaniasis (CL) in children. Miltefosine may be administered via in-home caregiver Directly Observed Therapy (cDOT), while patients must travel to clinics to receive MA. We performed a cost-effectiveness analysis comparing miltefosine by cDOT versus MA for pediatric CL in southwest Colombia.

**Methodology/Principle findings:**

We developed a Monte Carlo model comparing the cost-per-cure of miltefosine by cDOT compared to MA from patient, government payer, and societal perspectives (societal = sum of patient and government payer perspective costs). Drug effectiveness and adverse events were estimated from clinical trials. Healthcare utilization and costs of travel were obtained from surveys of providers and published sources. The primary outcome was cost-per-cure reported in 2015 USD. Treatment efficacy, costs, and adherence were varied in sensitivity analysis to assess robustness of results. Treatment with miltefosine resulted in substantially lower cost-per-cure from a societal and patient perspective, and slightly higher cost-per-cure from a government payer perspective compared to MA. Mean societal cost-per-cure were $531 (SD±$239) for MA and $188 (SD±$100) for miltefosine, a mean cost-per-cure difference of +$343. Mean cost-per-cure from a patient perspective were $442 (SD ±$233) for MA and $30 (SD±$16) for miltefosine, a mean difference of +$412. Mean cost-per-cure from a government perspective were $89 (SD±$55) for MA and $158 (SD±$98) for miltefosine, with a mean difference of -$69. Results were robust across a variety of assumptions in univariate and multi-way analysis.

**Conclusions/Significance:**

Treatment of pediatric cutaneous leishmaniasis with miltefosine via cDOT is cost saving from patient and societal perspectives, and moderately more costly from the government payer perspective compared to treatment with MA. Results were robust over a range of sensitivity analyses. Lower drug price for miltefosine could result in cost saving from a government perspective.

## Author summary

Cutaneous leishmaniasis (CL) is a tropical parasitic disease transmitted by sand flies that causes chronic skin and mucosal ulcers. Current standard of care therapy requires patients to travel to a clinic for twenty consecutive days for injections of meglumine antimoniate (MA). This may represent an economic burden, particularly for patients living far from healthcare services, especially children and their caregivers. We performed mathematical modeling to compare costs of the standard of care treatment with costs of miltefosine, an equivalently efficacious oral medication that allows pediatric patients to be treated at home under trained supervision of a caregiver. In our model, miltefosine led to substantially lower costs for patients and only slightly higher costs to the healthcare system. Importantly, the cost to society (combined patient and healthcare system costs) was lower for miltefosine compared to MA. Treatment of pediatric CL with miltefosine in the patient’s home could decrease overall cost of treatment, while diminishing the barriers and cost burden on patients, their caregivers, and society.

## Introduction

Cutaneous leishmaniasis (CL) is a neglected tropical disease primarily affecting poor, marginalized populations. Worldwide incidence of CL has been estimated at 1–5 million cases per year [1,2]. In Latin America, over 57,000 annual cases were reported on average between 2001 and 2013 [3]. In Colombia, 7,000–18,000 cases of CL are reported per year [4]. Of the 7,777 cases reported in 2015, 71.5% were from rural areas, and 17% were younger than 15 years of age [5]. A recent retrospective study of the clinical and epidemiological profile of pediatric CL patients in Colombia indicates that children are increasingly affected by the disease due to population movements and environmental factors bringing vectors into closer contact with domestic settings [6].

CL patients in rural areas face economic and geographic challenges to securing treatment, including travel through zones of armed conflict. Pediatric populations are expected to incur particularly high costs, as their caregivers must accompany them to clinic for treatment. Additionally, MA has been shown to have a higher rate of renal clearance in pediatric patients, contributing to lower systemic exposure and a higher failure rate than adults [7,8]. Finally, MA has been associated with infrequent but serious adverse reactions, as well as intolerance of the intramuscular route of administration [9].

Miltefosine, a well tolerated medication for CL, has been shown to be non-inferior to MA in a clinical trial in pediatric patients in Colombia [10]. Miltefosine is administered orally and could be given via caregiver directly observed therapy (cDOT) at the patient’s home.

Directly observed therapy (DOT) describes any protocol in which a trained observer watches medication administration to ensure compliance in order to avoid treatment failure and microbe resistance. Traditionally, this observer has been a health care professional, but protocols in which the observation is done in the home and by lay observers have been shown to be non-inferior in the case of tuberculosis [11], the disease currently most commonly treated by DOT protocols. In our study, cDOT implies education of a pediatric patient’s caregiver in a manner that ensures course completion and appropriate use, including safe medication usage, storage and disposal. While cDOT has not yet been implemented for CL treatment, the efficacy [12–17] and cost-effectiveness [12,16] of the cDOT model for the treatment of tuberculosis have been established in a variety of contexts, including in pediatric populations [15,17].

Caregiver administration could ease the economic burden of CL treatment on families of affected children, as well as improve access and adherence to treatment in remote areas. Despite evidence demonstrating efficacy, the pediatric formulation of miltefosine is not widely available in Colombia.

This study describes the relative costs of pediatric CL treatments with MA and miltefosine treatment for patients in southwest Colombia. This information is intended to guide policy makers, health ministries, and healthcare providers in countries with endemic CL.

## Methods

### Study design

We developed a cost-effectiveness analysis study using a Monte Carlo simulation model of CL treatment to examine the potential clinical and cost impact of miltefosine cDOT versus MA for a pediatric population with CL. The model incorporated data from multiple sources including public health databases [18–20], primary surveys, expert opinion, and published data in order to project cost to stakeholders over the course of CL treatment.

Simulated strategies were based on the interventional arms of the RCTs undertaken in Colombia and Brazil between 2007–2010 comparing the efficacy of intramuscular MA and oral miltefosine. Independent parameters were subjected to random assignment along assigned probability distributions in order to represent uncertainty and heterogeneity in these parameters.

The study was conducted with public health data from four municipalities in two recognized endemic areas in Colombia [21] which have active leishmaniasis treatment programs—the lowland Pacific coastal municipalities of Buenaventura and Tumaco, and the Andean Central Cordillera municipalities of Chaparral and Rovira. These sites are among the municipalities with greatest transmission of CL in Colombia [5].

The University of Chicago Institutional Review Board and the Ethics Committee of CIDEIM and Universidad Icesi approved and monitored the study.

### Perspective

Cost-effectiveness analyses were performed from patient, government payer, and societal perspectives [22]. The patient perspective included out-of-pocket costs assumed by patient caregivers during the course of treatment, such as transportation to clinics, meals outside the home, lodging, childcare, and medical supplies. The patient perspective excluded drug costs as these are publically covered. The government payer perspective included costs assumed by the Colombian healthcare system in the course of treatment such as drug costs, clinical medical supplies, and treatment associated with adverse events. The societal perspective combined the patient and government payer perspectives to estimate total cost associated with treatment.

Costs are reported in 2015 USD [23], and no discounting was applied, as the time frame of treatment was under one month. Sensitivity analyses were performed to test the stability of model outputs with variation of parameters.

### Outcomes

Primary outcomes were societal, patient, and government payer cost-per-cure for each treatment strategy. Cost difference is the MA cost-per-cure minus the miltefosine cDOT cost-per-cure. Cost neutrality is the point at which MA and miltefosine cDOT treatments incur the same cost-per-cure.

### Model structure

We employed a Monte Carlo simulation model (SimVoi v3.02 plugin for Microsoft Excel). Cases of clinically confirmed CL were simulated in a patient level probabilistic model in which unique patients entered the model and accrued costs to themselves and the government payer based on their treatment assignment, and left the model in either a cured or uncured state. Cure rate and adverse events during the course of treatment were modeled. Individuals failing treatment were not re-treated. Simulations of 100,000 patients were run for each potential intervention—meglumine antimoniate, miltefosine (availability of adult and pediatric formulations), and miltefosine (pediatric formulation only)—to ensure stability of results.

### Input data

Baseline characteristics of children ages 2–12 with diagnosed CL were obtained from the pooled National Public Health Surveillance System (SIVIGILA) public health records from the municipality of Tumaco, Nariño from January 2012-May 10, 2014 [18] and Chaparral, Tolima from March 7, 2003-December 7, 2011 [19]. The average age was 7.12 years and 49.7% of patients were female (Table 1). Patient weights were based on means and confidence intervals presented in the National Survey of the Nutritional Situation in Colombia in 2010 [24]. Weights were plotted on a normal distribution for each year of age for each sex.

**Table 1:** Cohort characteristics.

	Base Case Value	Distribution type	References
Cohort characteristics			
Mean age, years (SD)	7.12 (3.08)		[18]
Gender, % females	0.497		[18]
Mean weight, kg	20.2^a^	Normal	[10]

**SD**: standard deviation

^a^ mean weight from Rubiano et al. 2012 [10], information for weight in Monte Carlo model from National Health Survey [24].

#### Dosing

Dosages for simulated patients followed guidelines established by the Pan-American Health Organization and Colombian government [25,26]. MA was administered at 20mgSb/kg/day, and the number of necessary daily ampules was rounded up to next whole number and multiplied by the 20-day length of treatment regimen. Miltefosine was administered at 1.5–2.5mg/kg/day for the 28-day course of treatment. In the model, patients always received the highest dose they were eligible for under these guidelines. Treatment consisted of appropriate combinations of 10mg (pediatric formulation) pills and 50mg pills, or of multiples of only 10mg pills in the pediatric formulation only case. The number of daily capsules was multiplied by the 28-day length of treatment, and adherence was assumed to be 100%.

#### Treatment efficacy

The efficacies of the compared treatments were derived from the combined 2–12 year-old patients from the cohorts in the three available randomized clinical trials (RCTs). Intention-to-treat basis was utilized to maximize simulation of drug effectiveness. The cure rates were 66.7% (95% CI 64.2–69.2%) for MA and 73.9% (95% CI 71.1–76.7%) for miltefosine. Clinical cure was defined as complete re-epithelialization and absence of inflammatory signs for all lesions 6 months after initiating treatment. Beta-distributions were assigned to the efficacy data in the simulation. No benefits were assumed outside of the treatment to primary outcome timeframe. Because individual trials were powered for non-inferiority, equivalent cure rates were used as a base-case for the multi-variable sensitivity analyses [10,27,28].

#### Adverse events

The likelihoods of clinical adverse events (CAE) associated with MA and miltefosine treatments were obtained from the clinical trial reporting pediatric CAE [10]. Incidence of CAE was calculated for pediatric patients and sub-categorized by severity according to CTCAE 3.0 guidelines for adverse events (Table 2) [29]. Beta distributions were applied to the CAE incidence rates to reflect data uncertainty. Costs attributable to medical complications were considered according to the following scheme: CTC Grade 1 events required no treatment; CTC Grade 2 events required an outpatient medical visit; CTC Grade 3 events required a one-night inpatient hospitalization. No CAEs more severe than CTC Grade 3 were reported in the reviewed trials.

**Table 2:** Efficacy and toxicity of treatments.

	Base Case Value	Distribution	References
**Dosage**			
*MA*			
MA dosage (mg/kg/kg)	20		[25]
Days of MA treatment	20		[25]
*Miltefosine*			
Miltefosine dosage (mg/kg/day)	1.5–2.5		[25]
Days of Miltefosine treatment	28		[25]
**Efficacy of Treatments**			
***MA cure rate (95% CI)***	0.667 (0.641–0.691)	Beta	[10,27,28]
***Miltefosine cure rate (95% CI)***	0.739 (0.711–0.767)	Beta	[10,27,28]
Toxicity^a^			
*MA adverse event rate (CAE/patient)*	0.842	Beta	[10]
% CTCAE Grade 1	92.5%	Beta	[10]^a^
% CTCAE Grade 2	4.2%	Beta	[10]^a^
% CTCAE Grade 3	3.2%	Beta	[10]^a^
*Miltefosine adverse event rate (CAE/patient)*	0.754	Beta	[10]
% CTCAE Grade 1	97.5%	Beta	[10]^a^
% CTCAE Grade 2	2.5%	Beta	[10]^a^
% CTCAE Grade 3	0%	Beta	[10]^a^

**MA**: meglumine antimoniate; **CI**: confidence interval; **CAE**: Clinical Adverse Event; **CTCAE**: Common Terminology Criteria for Adverse Events.

^a^ Data from personal communication with L. Rubiano [10].

#### Drug prices

WHO negotiated pricing guidelines were utilized. The negotiated price of MA was $1.20 per 5-ml vial of 81 mg/ml. The price for miltefosine was $57.64-$69.61 for 56 50-mg capsules and $43.74-$50.03 for 56 10mg capsules [30]. MA prices were simulated on a lognormal distribution with a standard deviation of 10% of the negotiated value. Miltefosine prices were distributed along a normal curve truncated at the WHO price boundaries. Further variation in prices was considered in sensitivity analysis.

Costs of healthcare related to treatment of adverse events were assigned based on 2008 country data from the WHO-CHOICE 2011 database [31], and adjusted according to the Colombian health services consumer price index data from 2009 to April of 2015 [32].

#### Clinic and patient costs

Surveys were developed to query healthcare workers with experience treating pediatric CL patients on two domains—clinic costs and patient costs. Surveys were distributed by email or telephone to health care workers at medical institutions in Buenaventura, Chaparral, Rovira, and Tumaco that treat leishmaniasis in the southwest and Andean regions Colombia. Healthcare providers were asked to estimate costs incurred by their clinic during the course of treatment of pediatric CL. This included medical supplies, healthcare personnel, and facility costs.

Healthcare providers were also asked to estimate costs incurred by their patients’ families during the course of treatment including food, lodging, childcare, and medical fees. Patient transportation costs were estimated by healthcare providers based on knowledge of transportation costs between clinics and neighborhoods and villages where patients resided during treatment [18,19] (Table 3).

**Table 3:** Selected cost parameters.

	Base Case Value (USD, 2001–2015 average)	Range for Sensitivity Analysis	Distribution	References
**Drug Costs**				
Miltefosine 50mg (per box of 56)	63.78	50–200%	Lognormal	[30]
Miltefosine 10mg (per box of 56)	46.88	50–200%	Lognormal	[30]
MA (per ampule)	1.20	50–200%	Lognormal	[30]
**Clinic Costs**				
Personnel cost difference (Miltefosine cDOT—MA)	0			Assumed
Facility cost difference (Miltefosine cDOT—MA)	0			Assumed
Supplies for MA administration	24.87	50–200%	Lognormal	Survey Data
Proportion of clinics providing supplies (otherwise provided by patient)	0.45			Survey Data
**Healthcare System Costs**				
CTCAE Grade 1(mild toxicity/no healthcare visit)	0		Lognormal	[31,33]
CTCAE 2 (outpatient costs/visit)	31.81		Lognormal	[31,33]
CTCAE Grade 3 (inpatient cost/night)	102.36		Lognormal	[31,33]
**Patient Costs**				
Transport to Municipal Capital	10.36	50–200%	Lognormal	Survey Data
Local Transportation	35.07	50–200%	Lognormal	Survey Data
Meals	116.02	50–200%	Lognormal	Survey Data
Lodging	68.90	50–200%	Lognormal	Survey Data
Administration of Treatment	23.29	50–200%	Lognormal	Survey Data
Childcare	27.86	50–200%	Lognormal	Survey Data
Per Capita GDP/day productivity loss	26.71	0–100%	-	[34]

**MA**: meglumine antimoniate; **cDOT**: caregiver directly observed therapy; **CTCAE**: Common Terminology Criteria for Adverse Events; **GDP**: gross domestic product.

MA and miltefosine cDOT were considered to have the same staff and facility costs, a conservative assumption based on expert opinion of providers involved in care of CL patients. Costs of presentation to a clinic in a municipal center, including transportation, lodging, food, childcare, and treatment costs, were included once for patients in both groups for initiation of treatment. Patients treated with MA also accumulated the costs associated with attending a local clinic for the following 19 days for administration of the drug, while patients treated with miltefosine cDOT did not incur further costs for clinic attendance. Costs of supplies required to apply MA treatment were accrued by either the clinic or the patient, based on survey results describing who paid for this item. Miltefosine cDOT treatment did not imply any treatments supply costs in the base analysis, though it was considered in sensitivity analysis. In one-way sensitivity analysis, caregivers of patients treated with MA could incur lost-time costs, while caregivers of patients treated with miltefosine cDOT did not.

### Sensitivity analysis

One- and multi-way deterministic analyses of selected parameters were performed to assess impact on base case results. The impact of treatment adherence was estimated by variation in treatment efficacy. Drug costs were varied, as was lost-time cost up to 100% of the Colombian minimum daily wage [35] during the course of MA treatment. Model inputs obtained from the survey were varied, including costs for supplies, treatment cost, transportation, meals, lodging, and childcare.

A multi-way sensitivity analysis explored variations in the efficacy ratio of miltefosine cDOT over MA. The base case was considered to be equivalent efficacy (efficacy ratio = 1.00), and lower and upper bounds were derived from the upper 95% CI of miltefosine efficacy divided by the lower 95% CI of MA efficacy (efficacy ratio = 1.19) and a reciprocal lower bound was calculated by subtracting the reciprocal change from the baseline assumption (efficacy ratio = 0.81).

Cost-per-cure ratios (miltefosine cDOT/MA), in which 1 indicates equivalent cost for miltefosine cDOT and MA, <1 indicates cost savings with miltefosine cDOT, and >1 indicates MA cost saving, were calculated.

## Results

From the societal and patient perspectives, miltefosine cDOT was less costly compared to MA. Mean societal cost-per-cure for MA and miltefosine cDOT were $531 (SD±$239) and $188 (SD±$100) respectively, a difference of +$343. Mean patient costs per cure were $442 (SD ±$233) for MA and $30 (SD±$16) for miltefosine cDOT, a difference +$412. Miltefosine cDOT cost savings were driven by high costs associated with patient travel required for MA treatment, including transportation, lodging, childcare, and meals ($259 (SD±$144) for MA and $21 (SD±$12) for miltefosine cDOT).

From the government payer perspective, MA was less costly than miltefosine cDOT due to the higher drug cost of miltefosine (mean drug costs were $41 (SD±$16) for MA and $116 (SD±72) for miltefosine cDOT). Mean government payer costs per cure were $89 (SD±$55) for MA and $158 (SD±$98) for miltefosine cDOT, a difference of -$69 (Table 4).

**Table 4:** Results.

	Mean	SD
**Miltefosine cDOT**		
Patient	$30	$16
Government Payer	$158	$98
Societal	$188	$100
**MA**		
Patient	$442	$233
Government Payer	$89	$55
Societal	$531	$239
**MA—Miltefosine cDOT**		
Patient	+$412	
Government Payer	-$69	
Societal	+$343	

**SD:** standard deviation; **cDOT**: caregiver directly observed therapy; **MA**: meglumine antimoniate.

When availability of only the 10mg formulation of miltefosine was simulated, mean societal costs of $217 (SD±$93), mean patient costs were $30 (SD±$ 16), and mean government payer system costs were $187 (SD±$91) for miltefosine cDOT, with respective cost differences of +$344, +$412, and -$98.

### Sensitivity analysis

**Fig 1:**
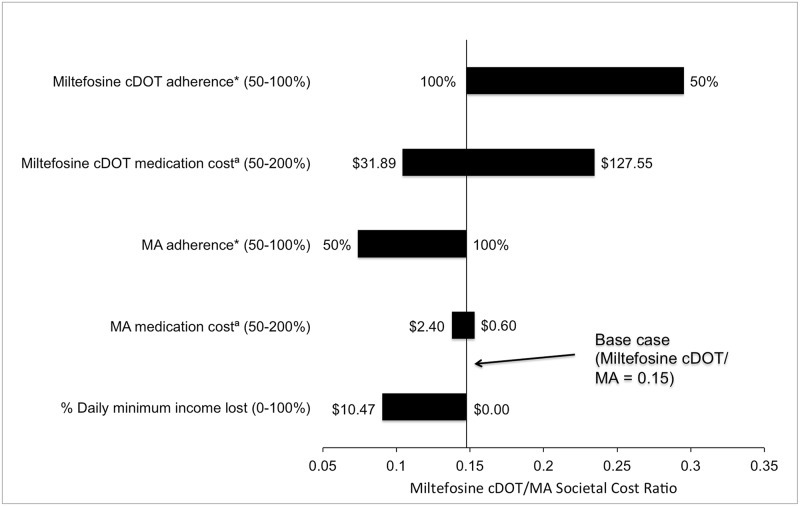
Tornado diagram of one-way sensitivity analyses of basic assumptions’ effects on miltefosine cDOT/MA cost ratio. Model parameters are listed on the vertical axis, with the range examined in sensitivity analyses in parentheses. The length of the horizontal bar demonstrates the impact of the changes in the parameter values on the cost ratio of miltefosine cDOT to MA. The solid vertical line indicates the estimated cost ratio of the base case. For example, an adherence rate of 100% was assumed for the miltefosine cDOT program in the base case. The cost ratio favored miltefosine cDOT for all simulated scenarios. *inverse of failure due to non-adherence, ^**a**^base case is WHO price. **cDOT:** caregiver directly observed therapy, **MA:** meglumine antimoniate, **WHO:** World Health Organization.

In one-way sensitivity analysis of baseline assumptions (Fig 1), miltefosine cDOT remained cost saving compared to MA across a wide variation of parameters, including drug adherence varies between 50–100%. Cost superiority of miltefosine cDOT was also maintained as miltefosine and MA drug prices were varied from 50–200% of WHO negotiated prices. Increased cost-effectiveness was seen with the inclusion of up to 100% of daily minimum wage loss for the caregiver during the course of MA treatment.

**Fig 2:**
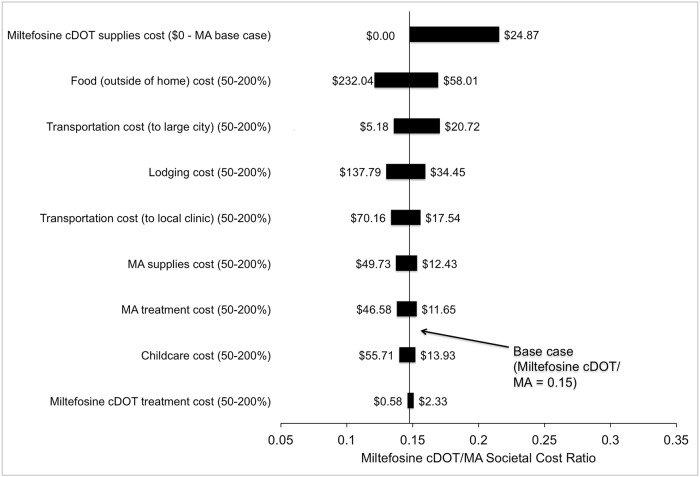
Tornado diagram of one-way sensitivity analyses of effect on miltefosine cDOT/MA cost ratio of parameters from survey data. Model parameters are listed on the vertical axis, with the range examined in sensitivity analyses in parentheses. The length of the horizontal bar demonstrates the impact of the changes in the parameter values on the cost ratio of miltefosine cDOT to MA. The solid vertical line indicates the estimated cost ratio of the base case. For example, a cost of $0 was estimated for the base case for miltefosine supply costs. The cost ratio favored miltefosine cDOT for all simulated scenarios. **MA:** meglumine antimoniate, **cDOT:** caregiver directly observed therapy.

One-way sensitivity analyses of cost parameters collected by survey instrument - medical supplies cost, treatment cost, food, lodging, childcare, and transportation—showed no change in cost-per-cure superiority when varied between 50–200% of mean collected data (Fig 2).

**Fig 3:**
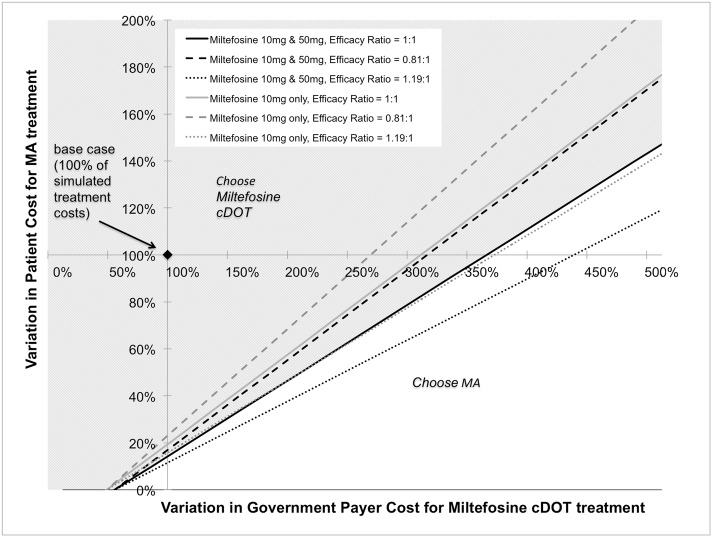
Multi-way sensitivity analysis of patient cost, healthcare system cost, drug formulation availability, and effectiveness. Cost to patients, the healthcare system, drug formulation availability, and effectiveness are varied simultaneously. The solid line represents equivalent efficacy of miltefosine and MA, varied over percent changes in MA-associated cost to patients and miltefosine cDOT-associated cost to the healthcare system. The dotted line represents the rightward shift in the boundary of cost-effectiveness when the effectiveness ratio of miltefosine to MA is increased to 1.19, as calculated from the upper bound of the miltefosine cure rate 95% CI divided by the lower bound of the MA cure rate 95% CI from the clinical trials. The hatched line represents the leftwards shift in the boundary of cost-effectiveness when the effectiveness ratio of miltefosine to MA is decreased to 0.81, calculated by subtracting the reciprocal change from baseline assumption. A diamond at the origin represents the mean case as calculated by the Monte Carlo model. **MA**: meglumine antimoniate; **cDOT**: caregiver directly observed therapy.

In multi-way sensitivity analysis, cost-per-cure ratio remained below 1 over a wide range of miltefosine cDOT-associated government payer costs and MA-associated patient costs (Fig 3). Miltefosine cDOT remained a cost-saving option from a societal perspective when MA-related patient costs were above 18% of the base case, and miltefosine cDOT-associated government payer costs was less than 355% of the base case. Stability in cost-per-cure ratio over these ranges was also demonstrated with the availability of 50 and 10mg formulations, as well as 10mg formulation only.

## Discussion

Our analytic model of treatment of CL in pediatric patients with miltefosine by cDOT versus current first-line MA treatment indicates that the miltefosine regimen is cost saving from a societal perspective. This result reflects considerably lower travel-associated costs for patients treated with miltefosine cDOT versus MA, a savings that exceeded the increased drug cost of miltefosine versus MA to the government payer. These results were robust across wide variations in parameters including adherence, medication efficacy, MA patient costs, miltefosine government payer costs, lost-time cost, adverse events costs, and direct patients costs. The availability of 50mg and 10mg formulations was associated with lower costs than availability of 10mg alone, but did not affect conclusions regarding cost superiority.

It should be noted that previous studies have estimated higher government payer cost-per-cure for MA, which may indicate further cost advantage of the miltefosine cDOT protocol. A study of an outbreak in Colombia estimated costs at $345 per cure; with MA drug costs of 300% of the cost assumed in our analysis [36]. Government payer cost-per-cure for MA in Guatemala and Peru has been estimated at $280 and $300, respectively [37,38]. The MA cost from these studies would exceed that estimated for miltefosine cDOT, making miltefosine cDOT cost-per-cure superior from the government payer perspective. An analysis of government payer cost-effectiveness of miltefosine and MA for adult CL patients in Colombia showed that miltefosine costs were nearly equivalent to MA costs [39]. However, no other analysis has focused on pediatric populations nor included patient and societal viewpoints.

Conversely, it should also be highlighted that our use of the WHO pricing guidelines may represent a low cost for miltefosine in Latin America. Despite these guidelines, procurement costs in practice have been observed to be considerably higher [39,40]. We emphasize that acquisition of competitive drug prices by government actors is a priority in providing miltefosine cDOT therapy in a cost-sensitive budgetary context. Acquisition of drugs for NTDs bought in the absence of a national public health program are likely to be higher than drug prices achievable though centralized high volume ordering [41–43], and as such, coordinated purchasing represents an opportunity for improvement of cost to the government payer. Additionally, pricing guidelines are subject to eventual renegotiation, in which case it is imperative that national, international, and non-governmental actors push for advantageous pricing of drug that carry significant benefits for marginalized patients.

Burden of disease studies have demonstrated that leishmaniasis and other NTDs cause significant detriment to the lives and livelihoods of patients and caregivers in endemic areas [44–48]. Our study highlights that decisions on public health matters by government payers should consider more than direct expense, and incorporate value added and costs avoided by different options, as well as the ethical mandate of protection of vulnerable populations. As in many low-resource settings, direct cost saving at the level of drug purchasing is attractive from a budgetary standpoint. However, a systemic perspective of costs of disease and treatment may reveal reversals of treatment cost-effectiveness superiority when patient and societal points of view are considered.

The findings of this study should be interpreted in the context of certain limitations. Firstly, effectiveness data for the treatment of CL is unavailable in Colombia and scarce among all countries of the region [49]. Secondly, the strict compliance conditions under which clinical trials are conducted do not represent the typical clinical experience with unsupervised treatment [50]. A 2014 Pan American Health Organization epidemiological report on the state of leishmaniasis indicates that only 31.6% of cases entered in the trans-national SisLeish surveillance system included clinical course [49]. The baseline assumption that adherence to medication was as observed in RCTs and did not vary between treatment regimens is a conservative assumption that may underestimate the benefits of oral miltefosine cDOT. Oral treatment is intended to lower barriers to care versus intramuscular injection of MA. Given that literature has estimated adherence to unsupervised miltefosine treatment for visceral leishmaniasis in Asia at 83% [51] to 95% [52], we consider a high degree of adherence under a cDOT program achievable. Nonetheless, adherence will be a crucial consideration during the design and implementation of a cDOT program.

Thirdly, modeling of costs, rather than direct costing of study participants was necessary due to the inclusion of a to-date theoretical cDOT protocol for miltefosine administration. The establishment and testing of specific protocols for a cDOT protocol remains a crucial step for this use of miltefosine. Among other concerns, the protocol must address re-administration of medication in cases of vomiting, the provision of specific education materials, implementation of methods to ensure adherence and adverse event accounting, and prevention of the use of the medication by household members of childbearing potential, due to miltefosine’s known teratogenicity [26,53].

Fourthly, susceptibility of distinct *Leishmania* species to particular drugs was not taken into account; however, current public health protocols do not identify species before initiation of treatment. A recent *in vitro* study of prevalent *Leishmania Viannia* species indicates high levels of susceptibility to both MA and miltefosine [54]. *L*. *panamensis* is the predominant strain in the area of the study [55] and has been shown to have good in vitro susceptibility to miltefosine [54]. While early tests of miltefosine indicated poor susceptibility of *L*. *braziliensis* [56], subsequent testing has found greater susceptibility in South American strains [57]. Local species and susceptibility patterns will be an important consideration in adapting miltefosine cDOT programs in other areas. Concern for the emergence of resistant strains as a result of poor adherence has been described in *L*. *Viannia species* [58], and necessitates that any forthcoming cDOT protocol ensure close monitoring to ensure continued drug efficacy.

Fifthly, variation in clinical course was simplified. Simulated patients did not experience spontaneous resolution of CL within the timeframe of the primary outcome and did not experience progression of their disease to disseminated or mucocutaneous leishmaniasis, since these variations in natural history would be expected to be comparable for equivalently efficacious drugs. Super-infection or other complications occurring during CL were not considered in the analysis. Rare but serious (CTCAE grades 4 and 5) complications were not included in the model, as none were experienced in the course of the trial from which modeling parameters were derived.

Sixthly, dosing parameters did not take into account re-dosing in the case of vomiting, or potential changes in pediatric dosing regimens given evidence from pharmacokinetic studies showing inadequate drug plasma levels under current dosing guidelines [59,60], although the costs of such cases may be extrapolated from sensitivity analysis of drug costs.

Finally, assessment of patient and clinic costs in remote, often conflict-stricken zones necessitated the use of surveys of healthcare provider to gain local perspectives of the costs to patient patients and caregivers. We believe that their assessment reasonably approximates the costs and logistics of treatment, including transportation costs, which were among the elements of highest impact in cost determination.

In summary, CL is a NTD causing significant morbidity and social stigma among marginalized pediatric populations. As new drugs are proven efficacious in treating this disease [10,27,28], opportunities for novel treatment protocols that reduce cost to both patients and national healthcare systems may be possible and merit further exploration. Our analysis shows that treatment of pediatric patients with a miltefosine cDOT protocol is cost saving from patient and societal perspectives across a range of assumptions, and efforts to reduce miltefosine pricing could ultimately lead to cost neutrality or cost savings from a government perspective. Development of such treatment programs represents a critical opportunity to improve treatment and outcomes for pediatric CL patients.

## Supporting information

S1 Spreadsheet Workbook Microsoft Excel document comprising Monte Carlo model set up, data referenced by the model, and survey answers. Requires SimVoi plug-in for modeling functionality.
(XLSX)
